# Clinical Pig Heart Xenotransplantation—Where Do We Go From Here?

**DOI:** 10.3389/ti.2024.12592

**Published:** 2024-02-02

**Authors:** David K. C. Cooper, Emanuele Cozzi

**Affiliations:** ^1^ Center for Transplantation Sciences, Massachusetts General Hospital and Harvard Medical School, Boston, MA, United States; ^2^ Department of Cardiac, Thoracic and Vascular Sciences, Padua University Hospital, Padova, Italy

**Keywords:** xenotransplantation, heart, initial clinical studies, outcomes, next steps

The recent sad death of Mr. Lawrence Faucette, the second patient to undergo pig heart transplantation at the University of Maryland at Baltimore (UMB), is a significant setback to the UMB program and, indeed, to all clinical attempts at organ xenotransplantation. However, such disappointments are to be anticipated when pioneering a completely new form of therapy.

The first patient to receive a human heart allotransplant, an operation carried out by Christiaan Barnard in Cape Town in 1967, sadly survived for only 18 days [1], far shorter than the 2-month’ survival of Mr. David Bennett, Sr, the first patient to receive a pig heart transplant at UMB [2]. However, Barnard’s second patient lived for a remarkable 19 months.

When new surgical treatments are introduced, e.g., open heart surgery, organ transplantation, most of the initial patients offered this novel high-risk treatment are desperately sick with no alternative therapy available to them. If they have a strong desire to live and sufficient courage, they are likely to accept any possible opportunity for prolongation of life, no matter how limited the chances of long-term survival.

This was certainly the situation in which Mr. Bennett and Mr. Faucette found themselves. Both had extremely poor myocardial function with left ventricular ejection fractions of 11%–12% (whereas the normal in a healthy adult should be >50%). For number of reasons, neither was deemed suitable for allotransplantation.

Mr. Bennett had been supported by extracorporeal membrane oxygenation (ECMO) for 6 weeks before undergoing heart transplantation and, as a result of being largely immobilized during this period and previously, was in an advanced state of debility that limited his recovery. Despite intensive physical therapy and good pig heart function for approximately 45 days, he was strong enough to get out of bed on only a single occasion during the 2 months that he survived.

His recovery was not helped by the fact that a dissection of his aorta at the site of the aortic cross-clamp at the time of the heart transplant, almost certainly associated with the fragility of his blood vessel walls because of his debility, required repair. To the surgical team’s credit, this was achieved successfully, but the complication resulted in renal failure, for which he required regular dialysis for the remainder of his life. The development of features suggestive of an abdominal infection or other intra-abdominal complication necessitated two laparotomies, undoubtedly contributing to his weakened state.

The very low levels of the immunoglobulins in his blood, again reflecting his prolonged debility, stimulated his medical advisors to administer intravenous immunoglobulin G (IVIg), which very likely contained anti-pig antibodies [3, 4] and may have been a factor in the development of the antibody-mediated rejection from which Mr. Bennett did not recover. In addition, the pig heart was found to harbor latent porcine cytomegalovirus (porcine roseolovirus, pCMV/pRV) whose reactivation and replication may have contributed to inflammation in the organ and to the patient’s demise [4–6].

Several aspects of Mr. Bennett’s care therefore needed careful reflection and some improvement to prevent complications in future patients. These included 1) removal of anti-pig antibodies from IVIg before its administration, and 2) a more sensitive test to determine whether the pig carried pCMV/pRV, but perhaps the most important lesson related to selection of the patient. If a patient is so debilitated that he or she is unlikely to recover full health, then possibly they should not be offered this form of therapy. When one considers the very checkered post-transplant course of Mr. Bennett, it is difficult to conclude that he benefitted in any way from the transplant though his family appreciated the extra time they could spend with him [7].

Because details of Mr. Faucette’s post-transplant clinical course have not yet been reported in the literature, we know much less about the factors that might have contributed to his demise, though dialysis was once again required for renal failure and rejection has been mentioned as the cause of death (after 42 days). This is particularly concerning as Mr. Faucette received an anti-CD154mAb-based immunosuppressive regimen (which is known to be more effective than an anti-CD40mAb-based regimen, which was the therapy that Mr. Bennett received), as well as increased complement-inhibitory drugs (a C1-esterase inhibitor followed by a C5 inhibitor, eculizumab). Unless there was a change in the medication schedule that has not been reported yet, if rejection was indeed the cause of graft failure, then there is cause for concern.

Numerous studies in gene-edited pig-to-nonhuman primate (NHP) models, including those at UMB, have provided encouraging data on pig heart [8–12] or kidney [13–15] survival when either an anti-CD40mAb or an anti-CD154mAb has formed the basis of the immunosuppressive regimen. In addition, there is now considerable *in vitro* evidence that strongly suggests that the immune hurdle will be significantly weaker when triple gene-knockout (TKO) pig organs (i.e., organs from pigs in which expression of all three known pig glycan xenoantigens has been deleted) are transplanted into humans than into NHPs [16, 17]. It is therefore disappointing and of concern that both patients might have lost their grafts from rejection.

In both patients, we presume that the presence of preformed anti-pig antibodies was low or had been excluded by pre-transplant testing, and so antibody-mediated rejection should only have occurred following the development of *de novo* anti-pig antibodies, suggesting inadequate immunosuppressive therapy.

In Mr. Bennett’s case, the factors that might have resulted in graft failure from rejection are more obvious than in the case of Mr. Faucette. In particular, it has been reported that anti-pig antibody concentrations remained low until postoperative day 47 when, following the administration of IVIg, a sharp increase of anti-pig IgG and, to a lesser extent, IgM was observed, possibly triggering a rejection response. Furthermore, mycophenolate mofetil therapy was discontinued due to pancytopenia from postoperative days 20–50 and instead the patient received tacrolimus from days 20 to 54. Indeed, his severely debilitated state may possibly have influenced the surgical team to reduce the intensity of immunosuppressive therapy to an inadequate level. In addition, the response to the presence of pCMV/pRV in the graft may have had a more detrimental effect on graft function than anticipated. (It has been well-documented that grafts from CMV-positive pigs fail earlier than those from pCMV/pRV -negative pigs [18, 19]).

However, all pioneering efforts are associated with errors and omissions, and it is easy to raise questions in hindsight. Without an initial effort, even if that effort is imperfect, no progress will be made. Hopefully, the causes of graft failure may become more clarified when data on Mr. Faucette’s post-transplant course are published.

But what can be done now by the UMB team and by others considering clinical gene-edited pig organ transplantation?

We suggest that the first consideration might be in determining whether pig kidney transplantation should be preferred over pig heart transplantation if only because, if the kidney fails or there are other complications, e.g., life-threatening infection, the pig kidney can be excised, all immunosuppressive therapy can be discontinued, and the patient returned to support by chronic dialysis [20]. At the present time, if pig heart xenotransplantation is justified (because neither allotransplantation nor mechanical support has been deemed possible), there can be no “Plan B”—if the heart fails, the patient will die.

Furthermore, in the experimental laboratory, numerous NHPs have been supported in a healthy condition by pig kidneys for more than a year, and for a maximum of almost 4 years in one case (Adams A, personal communication). In contrast, to our knowledge no NHP has survived while supported by an orthotopically-placed pig heart for >9 months, and failure has uniformly been from antibody-mediated rejection. The expectation that a gene-edited pig heart will support a patient for a prolonged period of time (in excess of a year) is therefore not currently supported by experimental data and may be overly optimistic at the present time. With the current moderately good results of mechanical device support in adults, it is difficult to justify bridging of an adult with a pig heart.

Instead, it has been proposed that xenotransplantation should first be employed as a method of bridging infants with complex life-threatening congenital heart disease, e.g., single ventricle physiology, until a suitable cardiac allograft becomes available [10, 21]. This approach has been suggested because 1) mechanical support devices are relatively rarely successful in infants and neonates, 2) the results of cardiac allotransplantation are better in this age group than of any other organ transplants in any other age group (in part because of their immature immune system and in part because partial or total thymectomy is commonly carried out to gain access to perform the operation), 3) the results of palliative surgery are mixed at best and considered unsatisfactory in many cases, and 4) bridging does not commit the recipient to a life-long dependency on a pig heart with all the “unknowns” with which this is currently associated.

If infants could receive a gene-edited pig heart transplant soon after the birth, this may well maintain life until a cardiac allograft becomes available, which in the United States is an average of approximately 4 months (with a waitlist mortality of 34%) [22, 23]. Even if the recipient becomes sensitized to pig antigens and produces anti-pig antibodies, the current (limited) evidence is that this would not be detrimental to the outcome of a subsequent cardiac allograft [24]. Furthermore, a successful xenograft would enable the baby to be taken home by the parents, whereas those supported by a mechanical device must remain in an intensive care unit for several months until an allograft becomes available.

As an increasing number of NHPs have been supported by pig hearts for 6 months or longer, this approach seems feasible and may be preferred to destination therapy in adult humans. The experience gained from bridging in infants could enable improvements in management to be made that lead eventually to successful destination therapy.

In summary, perhaps clinical pig xenotransplantation should at present best be directed towards kidney transplantation. Alternatively, bridging infants to cardiac allotransplantation represents an option that needs to be explored further.

## Data Availability

The original contributions presented in the study are included in the article/supplementary material, further inquiries can be directed to the corresponding author.
